# Ultrasound-Assisted Lumbar Interlaminar Epidural Dye Injection and Evaluation of Its Distribution by Anatomical Dissection

**DOI:** 10.3389/fmed.2019.00049

**Published:** 2019-03-12

**Authors:** Irina Evansa, Angelika Krumina, Anna Simonova, Viktorija Dzabijeva, Svetlana Fedorovica, Alla Hadunkina, Natalja Zlobina, Grigorijs Vabels, Eva Strike, Ludmila Viksna, Indulis Vanags

**Affiliations:** ^1^Department of Anesthesiology and Pain Medicine, Riga 1st Hospital, Riga, Latvia; ^2^Department of Anesthesiology and Intensive Care, Medical Faculty, Riga Stradins University, Riga, Latvia; ^3^Department of Infectology and Dermatology, Medical Faculty, Riga Stradins University, Riga, Latvia; ^4^National Forensic Medicine Center, Riga, Latvia

**Keywords:** cadaver, epidural space, injections, pain, lumbosacral region

## Abstract

**Background:** Epidural steroid injections are frequently used to treat lumbar radicular pain. However, the spread of a solute in the epidural space needs further elucidation. We aimed at assessing the distribution of green dye in the epidural space after lumbar epidural injection on cadavers.

**Methods:** We performed ultrasound-guided injections of green dye between lumbar vertebrae 4 and 5 in 24 cadavers. The cadavers were randomly divided into group A and B according to the volume of injected dye; 3 ml in group A (*n* = 13) and 6 ml in group B (*n* = 11). Accuracy of the needle insertion and patterns and distributions of the spread were compared between the groups. After local dissection, we examined the spread of dye in dorsal and ventral epidural spaces and presented the distribution as whole numbers and quartiles of intervertebral segments. Mann-Whitney U Test was used to compare distribution of dye spread between groups A and B. Wilcoxon Signed-Rank Test was used to compare the spread of dye in cranial and caudal direction within the group. We considered *P* < 0.05 as significant.

**Results:** Data were obtained from all 24 cadavers. Median levels of dorsal cranial dye distribution in groups A and B were 2 and 4 (*P* = 0.02), respectively. In the dorsal caudal−2 and 2, respectively (*P* = 0.04). In the ventral epidural space cranial dye spread medians were−0 and 2 in groups, respectively (*P* = 0.04). Ventral caudal spread was 0 and 1, respectively (*P* = 0.03). We found a significant difference between cranial and caudal dye distribution in group B (*P* < 0.05). In group A the dye spread was bilateral. In group B cranial and caudal dye spread was observed.

**Conclusions:** Ventral dye flow was observed in 50% of injections. Bilateral spread of dye occurred in 63%, and more often in group A. Cranial spread was slightly higher than caudal spread in group A despite a smaller injected volume, and significantly higher in group B following a larger volume.

## Introduction

Epidural steroid injections have been used to treat lumbar radicular pain syndrome since 1952 (1, 2). Since then, there have been concerns about both the technique of puncture, the injected drug volume and whether a blind injection technique is acceptable; or alternatively, whether visualization of the epidural space should be the “gold” standard. All these questions relate to the spread and absorption of steroid in the epidural space. Recent investigations have emphasized the importance of ventral spread to achieve a good clinical outcome (3). The anterior epidural space contains receptors for various pain triggering mediators, such as substance P, calcitonin gene-related peptide, c-fos as well as cytokines and other inflammatory products, explaining the rationale for a preferred drug delivery within the anterior epidural space (4, 5).

Currently, there is still a lack of direct observations of the distribution pattern of steroid solutions after epidural application. Some authors have used fluoroscopic guidance to distinguish between cranial and caudal distributions of X-ray contrast after epidural injection (6). However, from our point of view, fluoroscopic determination of contrast spread does not possess the same degree of accuracy as the direct measurement of dye distribution following injection into the epidural space. Therefore, the goal of the present investigation was to assess the distribution of green dye injected into the epidural space after median interlaminar puncture between lumbar vertebrae 4 and 5 on cadavers.

## Methods

The Ethical Committee of Stradins University, Riga, Latvia approved the study on January 14, 2014 [Protocol E-9(2)]. The study took place at the Department of Pathology at Stradins University Hospital, Riga, Latvia on January 2014.

**Figure 1:**
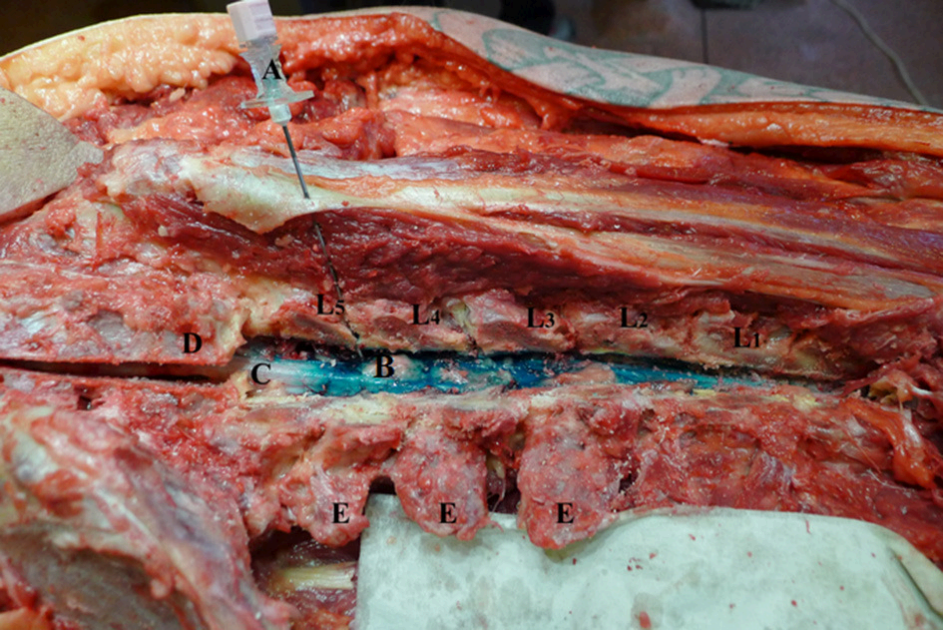
Touhy needle inserted in L4-L5 intervertebral level. L1–L5—lumbar vertebrae dissected at facet joints; **(A)** Touhy needle with bevel in dorsal epidural space at L4-L5 intervertebral space; **(B)** dye in dorsal epidural space; **(C)** spinal cord; **(D)** sacrum; **(E)** transverse process.

Following EC Directive 2004/23/EC standards, 24 non-embalmed adult human corpses were exposed to epidural injections of brilliant green dye, 10 mg/ml (Viride nitentis®, Riga Pharmaceutical Factory, Riga, Latvia) (7). The bodies were used within 15 h of death, which is a stated interval that avoids tissue changes (8, 9). The cadavers were randomly divided into a group A and B, according to the volume of dye injected: 3 ml in group A (*n* = 13) and 6 ml in group B (*n* = 11). All injections were performed in the lumbar epidural intervertebral space L4–L5, as documented with the tip of the Touhy needle in the interlaminar epidural space in Figure 1.

Inclusion criteria for the corpses were: age >18 years old without history of recent spinal trauma or surgery and absence of visible tissue lesion at the puncture site.

The bodies were placed in prone position on an autopsy table. Ultrasound imaging of the lumbar spine was performed prior to epidural needle insertion to visualize the interlaminar space and mark the injection site. We used a BK Medical Flex Focus ultrasonograph equipped with a 5.0 MHz curved array probe (Herlev, Denmark). The probe was oriented longitudinally and a parasagittal oblique view was used to mark the median injection point and interlaminar space by counting upwards from the sacrum line (10, 11). The transducer was angled toward the midline to visualize the epidural space, the spinal canal and the posterior aspect of the vertebral body (12–15). Subsequently, the probe was rotated to obtain a median transverse view of the lumbar spine, and the same structures were identified and marked for the midline injection approach. We found the acoustic window showing the best view of the epidural space and moved it to the middle of the screen. Whereupon the skin was marked lateral of the midpoint between both ends of the probe. We interpreted the intersection of four marked lines as the puncture site and noticed visually the angle of probe providing the best image of the interspace structures. The distance from the skin to the epidural space was also measured using a built-in caliper. Then, an 18-gauge 90 mm Tuohy-type epidural needle was advanced through the ultrasound-determined insertion point at an angle reproducing the direction of the ultrasound beam and depth of the epidural space during the pre-puncture examination. Puncture was performed using the loss of resistance technique (11, 12). Speed of the dye injection was approximately 1 ml s^−1^. An experienced doctor with abundant knowledge of spinal ultrasound succeeded with all procedures on the first attempt. Immediately after injection, an autopsy assistant performed bilateral incisions of facet joints from cervical to sacral levels. The Touhy needle was left in place in the epidural space during dissection in order to precisely define the intervertebral space, where the dye was injected. Firstly, the dye flow was assessed in cranial and caudal direction from the injection level in the dorsal epidural space. Then, the spinal cord was isolated and dissected free from adjacent tissues from the cervical to the sacral region to visualize dye spread in the ventral epidural space. An autopsy assistant, who was unaware of the injected dye volume, made spine dissection and determined dye spread and count of spinal levels.

Dye spread patterns were described as unilateral, bilateral, ventral and dorsal distributions (16). The ventral and dorsal spread was also described as being cranial or caudal from injection site and the number of lumbar spinal levels reached by the flow distribution was recorded. A level was defined on the spinal cord (after the *columna vertebralis* was dissected) from the dye injection point at L4–L5 level regarding it's cranial or caudal spreading, until the point where the dye distribution ended. Thus, if the dye reached intervertebral disc L3–L4 after an injection at L4–L5, it would be recorded as one cranial distribution level. If the dye reached the L5–S1 level (level of S1 *foramina sacralis*), one caudal level was recorded. The levels were rounded off in quarter intervals (the space between two spinal discs was divided in four equal parts horizontally to the spinal axis) in order to appreciate the levels when the dye stops somewhere between two spinal discs.

Data were analyzed with SPSS (SPSS® version 20, Chicago, IL). We used Shapiro—Wilk test to check the data for normal distribution. Not normally distributed data were analyzed with Mann-Whitney U Test for comparing A and B group dye spread and Wilcoxon Signed-Rank Test was used to compare within the group dye spread in cranial and caudal direction. We considered *P* < 0.05 as statistically significant.

## Results

**Figure 2:**
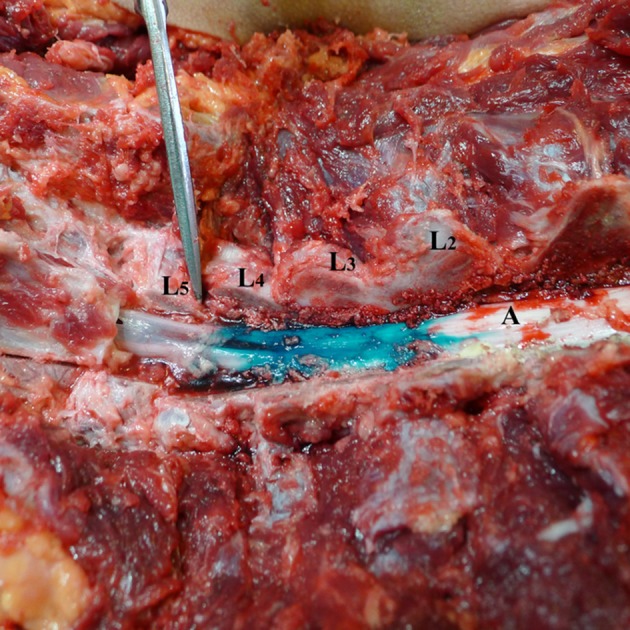
Spread of dye in dorsal epidural space after 3 ml volume injection (Group A). L1–L5—lumbar vertebrae dissected at facet joints; **(A)** spinal cord and dorsal epidural space with dye spread for 2.25 levels cranial.

**Figure 3:**
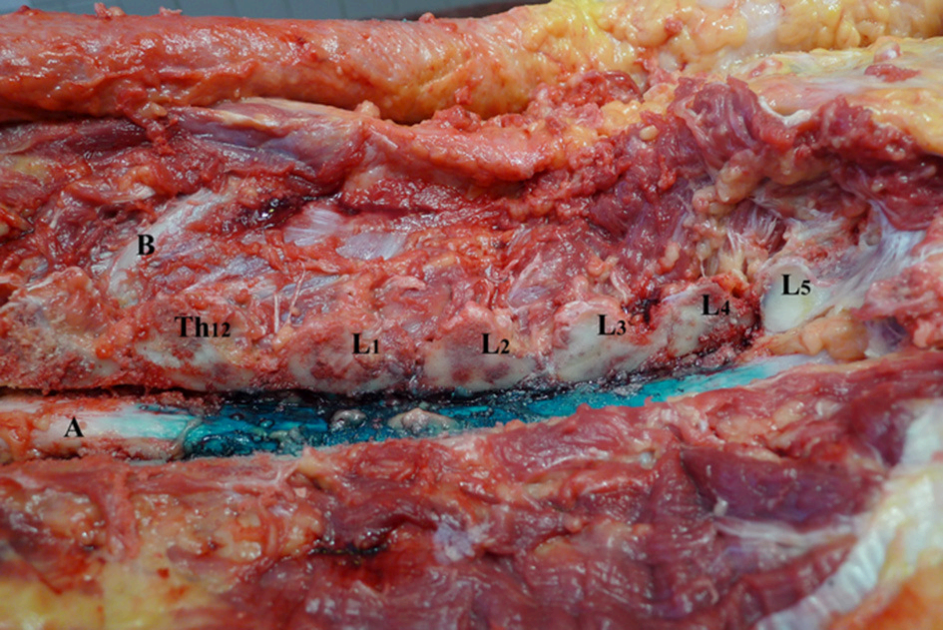
Spread of dye in dorsal epidural space after 6 ml volume injection (Group B). L1–L5—lumbar vertebrae dissected at facet joints; Th12—twelfth thoracic vertebrae; **(A)** spinal cord and dorsal epidural space; **(B)** 12th rib.

Data were obtained following dye injections at L4–L5 level in 24 cadavers, 15 men and 9 women. Average age at the time of death was 56 (range 32–80) years. Average weight and height were 83 (range 69–110) kg and 173 (range 156–190) cm, respectively. During ultrasonography, it was possible to view the epidural space, the spinal canal and the posterior aspect of the vertebral bodies of all 24 cadavers. We performed all punctures easily at the first attempt. Review of all dorsal epidural spaces after *columna vertebralis* dissection revealed a dye distribution in 100% of cases regardless of whether we had injected 3 ml or 6 ml of dye, as displayed in Figures 2, 3.

**Figure 4:**
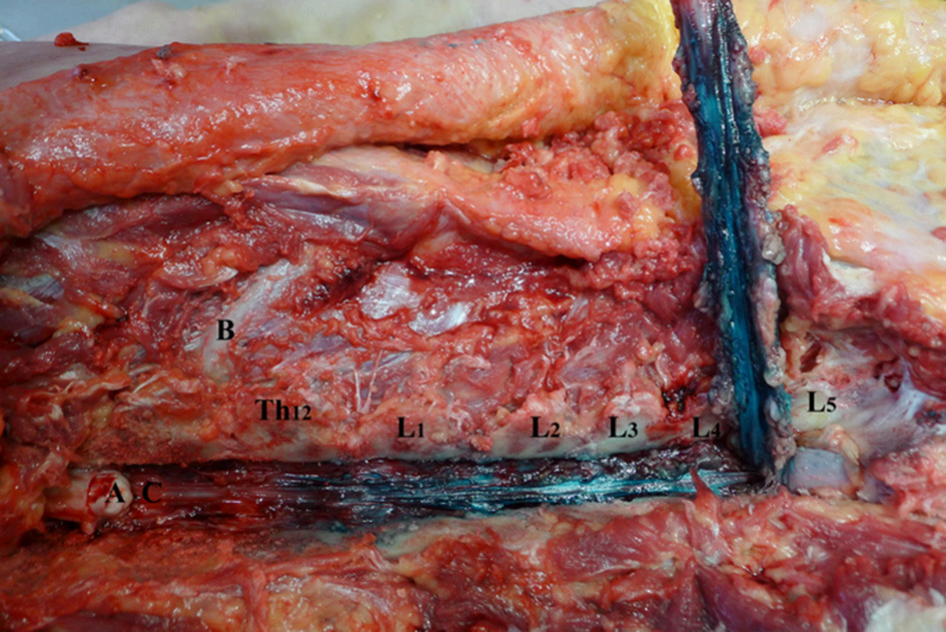
Spread of dye in ventral epidural space after 6 ml volume injection (Group B). L1–L5—lumbar vertebrae dissected at facet joints; Th12—twelfth thoracic vertebrae; **(A)** spinal cord; **(B)** 12th rib; **(C)** ventral epidural space.

Overall, in 50% of the cases ventral dye flow was witnessed. It was more frequently observed in group B compared to group A (*P* = 0.04; Table 1 and Figure 4).

**Table 1:** Direction of distributions of green dye with its patterns and levels of flow distribution from the injection site following all 24 injections.

	**Group A**	**Group B**	P^*^
**DIRECTION OF FLOW**			
Dorsal flow	13 (100%)	11 (100%)	*0.41*
Ventral flow	5/13 (38%)	7/11 (64%)	*0.04*
Bilateral flow	11/13 (85%)	4/11 (37%)	*0.05*
**MEDIAN NUMBER OF SEGMENT LEVELS WITH**			
**INTERQUARTILE RANGE**			
Dorsal cranial	2 (3–1)	4 (4–2)	*0.02*
Dorsal caudal	2 (2–1,5)	2 (2–2)	*0.04*
*P^#^*	*0.67*	*0.04*	
Ventral cranial	0 (2–0)	2 (4-0)	*0.04*
Ventral caudal	0 (0–0.5)	1 (2–0)	*0.03*
*P^#^*	*0.06*	*0.03*	

P^*^ Mann-Whitney U Test, exact one tailed P significant if < 0.05

*P^#^ Wilcoxon Signed-Rank Test, exact two tailed P significant if < 0.05*.

Median levels of dye distribution in dorsal cranial epidural space was 2 (IQR 3-1) and 4 (IQR 4-2) in groups A and B, respectively (U = 35.5; *P* = 0.02). Median levels of dye distribution in dorsal caudal epidural spaces in groups A and B were 2 (IQR 2-1.5) and 2 (IQR 2-2), respectively (U = 61.5 *P* = 0.04). Median level of dye distribution in ventral cranial epidural space was 0 (IQR 2-0) in group A and 2 (IQR 4-0) in group B (U = 43; *P* = 0.04). Ventral caudal spread was 0 (IQR 0-0.5) in group A and 1 (IQR 2-0) in group B (U = 43; *P* = 0.03).

Median dye distribution levels did not differ significantly between dorsal and ventral epidural spaces of group A (*P* = 0.06). In group B, we found a significant difference between cranial and caudal dye flows in both dorsal and ventral epidural spaces, respectively (Z = −2.33, *P* = 0.03; Z = −2.06, *P* = 0.04; Table 1). The cranial dye flow was significantly higher in this group.

**Figure 5:**
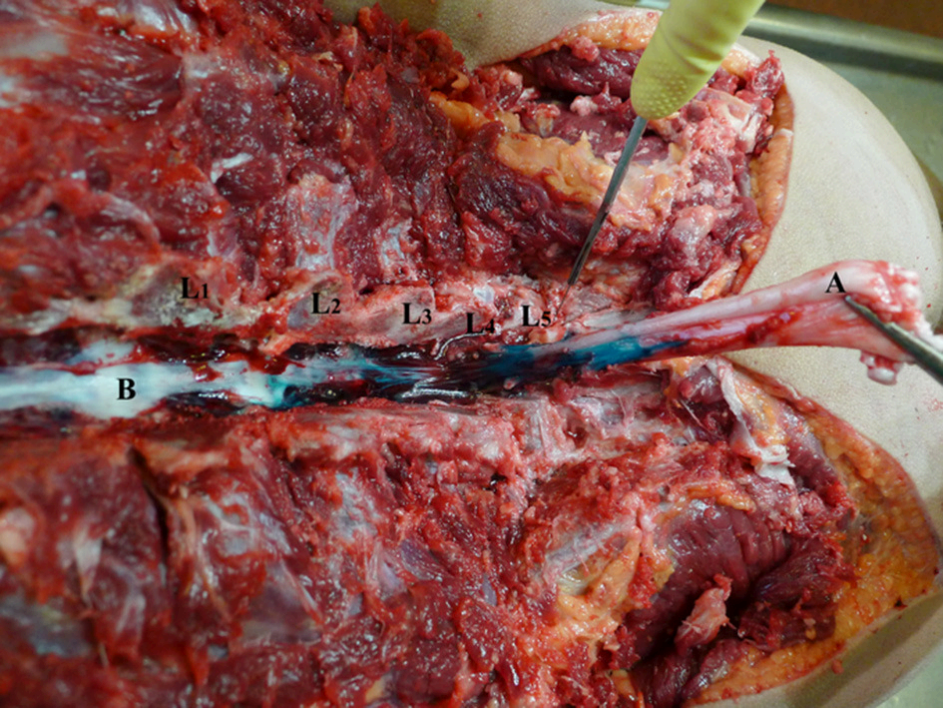
Unilateral dye spread in ventral epidural space after 3 ml volume injection (Group A). **(A)** dissected spinal cord at Th12-L1 level with dye spread on the left side. **(B)** ventral epidural space with unequal dye spread mostly on the left. L1–L5—lumbar vertebrae dissected at facet joints.

We observed bilateral dye flow in 15 cases. It occurred in 11 out of 13 cases (85%) in group A as compared to 4 out of 11 cases (37%) in group B (*P* = 0.05) (Figure 5).

## Discussion

The present study shows that green dye spreads cranially to the same levels in both dorsal and ventral epidural spaces. It also spreads in the caudal direction, but to a significantly lesser extent.

More extensive cranial spread of contrast, is consistent with the findings of major epidurographic studies (17–21). Not surprisingly, the dye distribution levels (cranial and caudal) seems to depend on the volume of injected dye. Thus, the levels are higher following larger injected volumes. We used smaller dye volumes (3 ml in group A and 6 ml in group B) in comparison with other investigators, who used 5 and 13 ml and observed similar results. The latter observations indicate that an appropriate spread might be obtained despite the use of smaller drug volumes (9, 19, 20, 22).

Weil and coworkers reported a ventral spread of dye in 47% of the cases after epidural injections of 5 ml dye in 114 patients, which is consistent with the findings of the present investigation (50%). In the same study, the investigators reported dye spread to more than 1 level caudally in 76% of the cases, whereas median dye spread caudally in the dorsal epidural space was 2 levels in both groups, as reported above (18). Thus, despite we used smaller dye volumes we observed greater distribution of dye.

Bilateral flow distribution was observed in 85 % of the corpses in group A. Interpretation of these data are demanding, since bilateral spread can be achieved even when using a smaller volume of dye, which is crucial when referring to patients with bilateral radicular pain.

A unilateral spread in 38% of the cases might be due to needle displacement. We assume that the needle was inserted laterally to the borders of the spinous process. Weil and co-workers also discussed this issue (18). If the needle tip was inadvertently placed laterally to the borders of the spinous process, unilateral spread occurred in 76% of the cases. Botwin et al. using a medial interlaminar approach and fluoroscopic control of the needle during puncture, observed unilateral flow in 84% of the cases (19). In contrast, our injections resulted in unilateral flow in less than half of the cases (*n* = 9). This could be related to more optimal control of needle insertion by using pre-puncture spine ultrasound examination. Alternatively, it could also be because we made the assessment after the dissection, thereby giving the dye more time to spread bilaterally in the dorsal epidural space. This also correlates with the findings of Hogan and co-workers, who performed cryomicrotomy 1 h after the injections (9, 19). An epidural contrast flow computerized tomography study by Paisely et al. revealed a bilateral pattern of contrast flow distribution 15 min after the injection in all the subjects (20, 23). Their findings warrant further investigations of dye spread in epidural space at different time points after the injection.

It has been shown that an interlaminar approach is safer for successful epidural steroid injections (17, 24–26). Cryomicrotome epidural space studies have revealed that there are no active boundaries for distribution of solutions in epidural space (27, 28). Consistent with the present study, the latter investigators used a median interlaminar approach for epidural puncture, injected 13 ml of dye and observed dye flow bilaterally and in ventral epidural space in 100% of cases (9). In our study, we used lower dye volumes and observed ventral flow in 38 % in group A and in 64% in group B. The latter results may be explained by application of smaller volumes of dye or a larger time difference between injection and sectioning of the spine. However, our data are consistent with a variety of epidurographic studies, in which imaging was performed immediately after contrast administration. In these studies investigators observed ventral contrast flow distributions in 36% to 90% of cases (17–20, 29).

The present investigation, in its capacity of being an observational study on cadavers, has several limitations. For obvious reasons, some aspects may differ from studies made with CT myelography or fluoroscopic epidurography on living subjects. It is possible that during dissection some disturbance of the dye fixation could have taken place. Apparently, the difference between findings in living subjects and cadaveric studies corresponds with the longer time needed to perform the observations. Another influencing factor could be loss of muscle tone in corpses as opposed to movement in paraspinal muscles, which could alter the pressure in the epidural space as well as in the dural sac. *In vivo*, the latter is supported by cerebrospinal fluid pressure, but is unsupported in cadavers. Inevitably, the tissue textures may change, because of temperature lowering. Even though we did not use any living subject to validate our data, the principal findings agree closely with similar *in vivo* studies. We also admit that the viscosity of dye and the speed of injection also could cause minor changes in the distribution pattern. For now, data are lacking as to whether these factors correlate significantly with the spread of steroid solutions in the epidural space. For obvious reasons, one of the major limitations of our study is the lack of information on low back pain symptoms and on the epidural space itself before intervention. In addition, rigor mortis and eventually some pathological conditions might have altered the distribution of solution in epidural space.

## Conclusion

An evaluation of dye distribution in epidural space shows that cranial spread is high in both dorsal and ventral spaces. Caudal spread of dye is significantly lower than cranial spread. There is also a tendency for the dye to spread higher cranially and lower caudally after injection of a larger volume of dye.

The pattern of observed cranial flow of dye should be studied clinically to determine if it affects outcome of therapy in patients. Further analyses including *in-vivo* studies are needed to demonstrate the effects of our findings on outcomes.

## Data Availability

All datasets generated for this study are included in the manuscript and/or the supplementary files.

## Author Contributions

All authors have contributed equally to the design of the study, to the analysis and interpretation of the results. IE, AS, VD, AH, SF conceived the study. IE included cadavers into the study and got agreement of participation, as well as continued to collect the data. IE was responsible for cadavers' recruitment and epidural injections. GV, NZ, IV, ES, AK, LV were responsible for critical revision of the article, final approval of the article and agreement to be accountable for all aspects of the work. All authors read and approved the final manuscript.

### Conflict of Interest Statement

The authors declare that the research was conducted in the absence of any commercial or financial relationships that could be construed as a potential conflict of interest.
